# Beam quality and the mystery behind the lower percentage depth dose in grid radiation therapy

**DOI:** 10.1038/s41598-024-55197-0

**Published:** 2024-02-24

**Authors:** Amir Hossein Karimi, Indra J. Das, Nahid Chegeni, Iraj Jabbari, Fatemeh Jafari, Ghazale Geraily

**Affiliations:** 1 Department of Medical Physics and Biomedical Engineering, School of Medicine, Tehran University of Medical Sciences, Tehran, Iran; 2 Radiation Oncology Department, Cancer Institute, Imam-Khomeini Hospital Complex, Tehran University of Medical Sciences, Tehran, Iran; 3 Department of Radiation Oncology, Northwestern Memorial Hospital, Northwestern University Feinberg School of Medicine, Chicago, IL USA; 4 Department of Medical Physics, School of Medicine, Ahvaz Jundishapur University of Medical Sciences, Ahvaz, Iran; 5 Department of Nuclear Engineering, Faculty of Physics, University of Isfahan, Isfahan, Iran

**Keywords:** Grid therapy, Small field dosimetry, Spectrometry, Monte Carlo, Linac, Biophysics, Oncology, Physics

## Abstract

Grid therapy recently has been picking momentum due to favorable outcomes in bulky tumors. This is being termed as Spatially Fractionated Radiation Therapy (SFRT) and lattice therapy. SFRT can be performed with specially designed blocks made with brass or cerrobend with repeated holes or using multi-leaf collimators where dosimetry is uncertain. The dosimetric challenge in grid therapy is the mystery behind the lower percentage depth dose (PDD) in grid fields. The knowledge about the beam quality, indexed by TPR_20/10_ (Tissue Phantom Ratio), is also necessary for absolute dosimetry of grid fields. Since the grid may change the quality of the primary photons, a new ${\mathbf{k}}_{\mathbf{q},{\mathbf{q}}_{0}}$ should be evaluated for absolute dosimetry of grid fields. A Monte Carlo (MC) approach is provided to resolving the dosimetric issues. Using 6 MV beam from a linear accelerator, MC simulation was performed using MCNPX code. Additionally, a commercial grid therapy device was used to simulate the grid fields. Beam parameters were validated with MC model for output factor, depth of maximum dose, PDDs, dose profiles, and TPR_20/10._ The electron and photon spectra were also compared between open and grid fields. The d_max_ is the same for open and grid fields. The PDD with grid is lower (~ 10%) than the open field. The difference in TPR_20/10_ of open and grid fields is observable (~ 5%). Accordingly, TPR_20/10_ is still a good index for the beam quality in grid fields and consequently choose the correct ${\mathbf{k}}_{\mathbf{q},{\mathbf{q}}_{0}}$ in measurements. The output factors for grid fields are 0.2 lower compared to open fields. The lower depth dose with grid therapy is due to lower depth fluence with scatter radiation but it does not impact the dosimetry as the calibration parameters are insensitive to the effective beam energies. Thus, standard dosimetry in open beam based on international protocol could be used.

## Introduction

Megavoltage grid therapy has been proposed for bulky palliative tumors with favorable outcomes. The treatment field is divided into smaller fields through a block called a grid^1,2^. Bulky tumors are radioresistant^3^ and require higher dosage that produces a high degree of complication rate to normal tissues. Mohiuddin et al.^4^ showed that small narrow pencil beam irradiation can help palliate the tumor. The success of this technique was based on the fact that small volumes of tissues could tolerate high doses of radiation which was attempted in a pilot study^4^. This was further extended with megavoltage beams and a grid made out of cerrobend whose characteristics have been reported by Reiff et al.^5^. The main mechanism of cell death and success observed in bulky tumors is not well understood, but it is considered to be the bystander effect^6^. Nevertheless, it is still an active research in the field of radiobiology. This has been eluded recently with some publications^7,8^.

Grid therapy of bulky tumors larger than 8 cm in diameter, which did not respond well to conventional doses was attempted^9^. The achievement is the ability to deliver a single high fraction of the dose (10–20 Gy) without significant complications to the normal tissue surrounding the tumor^10^. The simplest approach for grid therapy is to divide the beam into several small circular fields by installing a block called the grid on the linac. External devices (brass grid collimators) are relatively cheap, but its usage require non-traditional approach. Various techniques have been in use or proposed for Spatially Fractionated Radiation Therapy (SFRT) including grid collimator, lattice therapy, multi-leaf collimators (MLCs), VMAT, and proton pencil beam scanning (PPBS) approaches^11–19^. Although MLC-based SFRT improves normal tissue sparing^20^, grid collimator-based SFRT delivers the dose with a consistent heterogeneity^7^.

Despite the well-performed research on the clinical achievements of grid therapy^9,10,21,22^, the dosimetric evaluation of this modality has not yet been investigated completely. Grams et al.^23^ discussed calibration and commissioning but failed to address the fundamental part of the dosimetry. The available dosimetric studies only addressed the measurement of percentage depth dose (PDD), dose profile, and estimation of treatment efficiency^5,7,23–25^. An interesting point is that the PDDs, under central hole in grid fields, are significantly lower than in open field^5,25^. Intuitively, it is expected that the role of the grid may be beam softening along the central axis of the beam which is counter argument to the publication by Verhaegen et al.^26^. This raises the question of beam calibration using standard dosimetry protocols^27,28^. If small-field protocols should be used, the proper correction factors should be used as described in the literature^29,30^, but there are no guidelines or literature on this topic.

Generally, there are two non-investigated scenarios about the effect of the grid on the quality of the photon beam. The first one is beam hardening; it means the grid removes the low-energy photons coming from the linac head. Such a scenario was studied by Geraily et al.^31^ to investigate the effect of physical wedges on the beam hardening. It was found that physical wedges have more significant effects on beam quality for 6 MV photons compared to 18 MV photons. The second one is that the grid produces photon and electron scattering and subsequently making the beam softer. This scenario has been looked at the context of stereotactic cones with a single hole. Verhaegen et al.^26^ used MC simulation and showed that small cones have effective higher energy compared to large cones. By the way, the challenge is the mystery behind the lower PDD in grid fields. Definitely, it is related to the change in photon and electron spectra in the water. Given that no scientific evidence is available in the literature to explore the mystery behind the lower PDDs in grid fields, this study was undertaken to study the photon as well as electron spectra in grid fields. Using these spectra, the unknown contribution of primary and scattered photons to the resultant depth dose under the grid field's central hole can also be determined.

Recently, it was recommended several areas of improvement for the application of SFRT in cancer treatments because SFRT is still a non-routine radiotherapy modality, and more support is requested in understanding the radiobiology, commissioning of TPS, and dosimetric indexes for this technique^32^. The knowledge about the beam quality, indexed by TPR_20/10_ (Tissue Phantom Ratio), is also necessary for absolute dosimetry of grid fields. Since the grid may change the quality of the primary photons, a new ${k}_{q, {q}_{0}}$ (beam quality correction factor) should be probably requested for absolute dosimetry of grid fields. Accordingly, in this study, the change in TPR_20/10_ in grid fields was also investigated in comparison with open fields using MC simulation.

## Materials and methods

### Geometry of simulation

**Figure 1:**
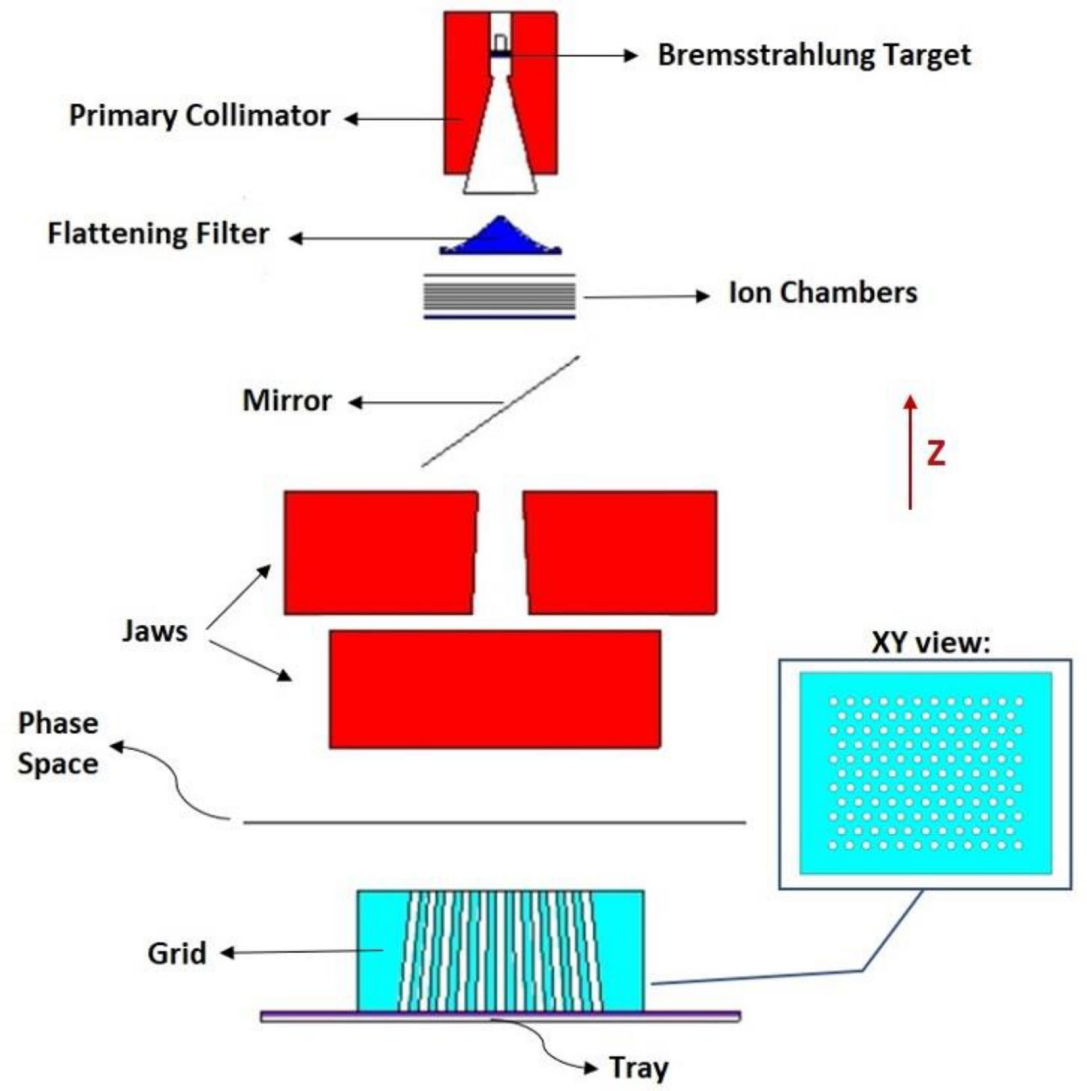
The main parts of a typical 6 MV linac (Varian 2100 C/D) and the grid block simulated by MCNPX.

In this study, the main components of a typical medical linac in 6 MV mode were considered to be simulated using MCNPX 2.7.0 Monte Carlo code^33^. For this purpose, the main parts of Varian 2100 C/D accelerator, including the target, flattening filters, primary collimator, ionizing chamber, mirror, and jaws were modeled based on the data released by Varian Corporation (California, Varian Medical Systems, USA). Additionally, a commercial grid therapy device made by Dot Decimal company (Sanford, FL 32771, USA) was used to simulate the grid-fields and mounted on the Perspex tray. The grid consists of 127 holes arranged hexagonally. The conical holes follow the divergence of the beam and produce circular fields with a 1.0 cm diameter at the isocenter. It is noteworthy that the center-to-center distance of the circular fields at the isocenter is 2 cm. This grid can irradiate a maximum field size of 25 × 25 cm^2^ at the isocenter. The pattern for simulation of grid was extracted from the literature ^34,35^. In Fig. 1, the scheme of simulated geometry was shown in detail. All Monte Carlo calculations were carried out on 32 paralleled cores owned by 4 computing servers (FT48, Tyan, Taiwan). Each server was equipped with 128 GB RAM and 4 AMD Opteron CPUs (4 × 16 = 64 cores). The energy cut-off for photon and electron transport was 0.01 MeV and 0.5 MeV, respectively.

### Model validation

The MC simulation was validated precisely with the measured data from the linac. Generally, the validation process of the MC simulation aims to evaluate the simulated geometry, correct selection of cross-section libraries for the physical interactions of radiation with matter, and finally beam adjustment. In this study, cross-section libraries of MCPLIB 04 (Rayleigh and Compton scattering, pair production, and the photoelectric effect) and EL 03 (elastic and inelastic collision, Bremsstrahlung, and annihilation) were applied for photon and electron interactions, respectively.

For tuning the electron beam impinging on the target, spatial and energy distributions of the electrons were considered to be Gaussian. Different energy peaks for the electrons were tested to fit the calculated PDD curve with those measured inside the water by the CC13 ionization chamber (IBA group, Louvain-La-Neuve, Belgium). In the following, the FWHMs (full width at half maximum) for the energy and spatial distributions of the electrons were optimized by comparing calculated dose profile curves with those obtained by the measurement. The comparisons were carried out using the gamma-index function with the criteria of 3 mm for distance to agreement and 3% for dose difference as shown by Low et al.^36^. A user-friendly code for gamma-index calculation was also developed in MATLAB software and attached to the Supplementary Material Section (S1 File). The electron beam is considered to be properly optimized if the gamma index analysis is passed for ~ 100% of the calculated points. The validation process was carried out step-by-step for open fields of 10 × 10 cm^2^ and 20 × 20 cm^2^.

After tuning the electron beam, a phase space file was produced on the top of the grid (Fig. 1). This trick efficiently reduced the time running MC codes. For this purpose, 2 × 10^10^ electrons impinging on the Bremsstrahlung target were traced and using the SSW (surface source write) card, nearly 52 × 10^6^ and 161 × 10^6^ photons were stored for 10 × 10 and 20 × 20 cm^2^ field sizes, respectively. In the next simulations (Sects. 2.3.3–7), while the linac head was still kept in the simulations, using the SSR (surface source read) card, photon transport was started from the phase space instead of electrons impinging on the Bremsstrahlung target.

### Dosimetric parameters: grid field vs. open field

In this study, the dosimetric parameters were calculated for both open and grid fields of 10 × 10 cm^2^ and 20 × 20 cm^2^. To investigate the effect of block components on the dosimetric parameters of grid fields, two types of grid blocks were used in the simulations. The first grid was made of brass (37% Zn and 63% Cu) and the second grid was made of cerrobend (10% Cd, 13.3% Sn, 26.7% Pb, and 50% Bi).

#### PDD and output factors

To calculate the PDD curves, a 50 × 50 × 50 cm^3^ water phantom was simulated at 100 cm SSD (source to surface distance). Using cylindrical mesh tally type 1 (cmesh1: e pedep), the absorbed dose of photons was calculated inside the cylindrical voxels arranged along the central axis of the photon beam. Each voxel is dimensioned by 4 mm in radius and 2 mm in height. Finally, PDDs were obtained using dividing the calculated doses by their maximum values. The depth of the maximum dose was also recorded for the next analyses.

The output factors were determined also by:

1 $${\mathrm{Output\,Factor}}_{\mathrm{ a}\times {\text{a}}}=\frac{{{\text{D}}}_{{\text{a}}\times {\text{a}}}}{{{\text{D}}}_{10\times 10}}$$

here, ${{\text{D}}}_{10\times 10}$ is the reference dose at maximum depth (${{\text{d}}}_{{\text{max}}}$) in the open field of 10 × 10 cm^2^ and ${{\text{D}}}_{{\text{a}}\times {\text{a}}}$ is photon absorbed dose at ${{\text{d}}}_{{\text{max}}}$ for specific field side of a, which can be open or with a grid.

#### Dose profiles

Using rectangular mesh tally type 1 (rmesh1: e pedep), the absorbed dose of photons was calculated in rectangular voxels (0.5 × 0.5 × 0.1 cm^3^) located at a depth of 10 cm inside the water phantom. The SSD was considered to be still 100 cm. Finally, dose profiles were obtained using dividing the calculated doses by the dose at the central axis.

#### *TPR*_*20/10*_

As mentioned before, the grid has the potential to change the quality of the photon beam. To address this challenge, TPR_20/10_ index was compared between open and grid fields. Considering the definition of the TPR_20/10_, the water phantom was simulated in two different conditions. The first, in 80 cm SSD to find the absorbed dose of photons at 20 cm depth, and the second, in 90 cm SSD to find the absorbed dose of photons at 10 cm depth. Dividing the first by the second results in TPR_20/10_. Here, dose calculations were performed at a cylindrical voxel with a 0.4 cm radius and 4 mm height using cylindrical mesh tally type 1. For the open field, the calculated TPR_20/10_ was compared with measurement under the same situation.

#### Photon spectra

Photon spectra were calculated at depths of 1.5, 5, and 10 cm inside the water phantom at 100 cm SSD. To this aim, the energy range of 0.01–6.02 MeV was linearly divided into 100 equal bins, and the F4: p tally was used to calculate the photon fluence in each bin. The calculations were performed at the cylindrical voxel with a 4 mm radius and 2 mm height. In this study, photon spectra were separately calculated for primary, scattered, and total photons. The term “primary photons” is arbitrarily attributed to the photons coming directly from the linac head without any interaction with the grid, perspex tray, or water. The FT and FU cards in MCNPX 2.7.0 were employed to tag the photons and categorized them to scattered and primary. Finally, the average energy of photons for both primary, scattered, and total photons was calculated from using Eq. (2):

2 $$\overline{E }=\frac{\sum_{{\text{i}}=1}^{{\text{j}}=100}{{\text{E}}}_{{\text{i}}}\cdot\upphi ({{\text{E}}}_{{\text{i}}})}{\sum_{{\text{i}}=1}^{{\text{j}}=100}\upphi ({{\text{E}}}_{{\text{i}}})}$$

where Φ (${{\text{E}}}_{{\text{i}}}$) is the calculated fluence related to the *i*-th energy bin and ${{\text{E}}}_{{\text{i}}}$ is the middle energy point in the *i*-th energy bin.

#### Dose components

Contribution of primary and scattered photons is of interest especially in grid fields. For this purpose, FT and FU cards in MCNPX 2.7.0 were used again to tag the photons. Remember that primary photons are the same photons coming directly from the linac head without any interaction with the grid, Perspex tray, or water. Photon dose and its component were calculated at cylindrical voxels with a 4 mm radius and 2 mm height along the beam axis under the central hole. The calculations were performed at different depths of 1.5, 5, and 10 inside the water. Finally, the results were compared with the open fields. To make the comparison more sensible, calculated doses were divided by the maximum value (total dose in open field of 20 × 20 cm^2^).

#### Photon fluence in air

To understand the attenuation from the grid, the total fluence of photons reaching the water surface was calculated using F4: p tally on a typical 10 × 10 cm^2^ field for both open and grid fields. The calculation was carried out inside a 50 × 50 × 0.1 cm^3^ cube located below the grid at a distance of 35 cm far away from the isocenter.

#### Electron spectra

To this end, using the energy card in MCNPX, the energy bin of 0.5–6.02 MeV was linearly divided into 92 equal bins, and the electron fluence (F4: e tally) was scored in 60 keV bins. The electron fluence was calculated in each bin at a cylindrical voxel with a 4 mm radius and 2 mm height at depths of 1.5, 5, and 10 cm inside the water phantom located at 100 cm SSD. Finally, the average energy of electrons was calculated via Eq. (2) (consider j = 92). Table 1 summarizes the type of tally, field size, voxel dimensions, and calculation depths used in this study.

**Table 1:** Summary of the calculation setup.

	PDD	Profile	Photon spectra	Electron spectra
Filed size (cm^2^)	10 × 10 and 20 × 20			
Tally type	cmesh1: e pedep	rmesh1: e pedep	F4: p	F4: e
Voxel dimensions	4 mm radius 2 mm height	0.5 × 0.5 × 0.1 cm^3^	4 mm radius 2 mm height	4 mm radius 2 mm height
Depth (cm)	0–30	10	1.5, 5, and 10	1.5, 5, and 10

### Unit conversion from "Per Electron-history" to "Per MU"

MCNPX reports the results based on 1 source particle, here it means 1 electron impinging on the Bremsstrahlung target. However, it is prudent to analyze the results based on 1 Monitor Unit (MU) of radiation (1 MU equals 1 cGy dose delivered to the depth of maximum dose under standard conditions, 100 cm SSD and 10 × 10 cm^2^ field size). So, to convert the unit of photon fluence (electron fluence, as well) from 1/cm^2^/electron-history to 1/cm^2^/MU, the data in the MCNPX’s output file was multiplied by the number of electron-histories required to deliver 1 MU of radiation.

## Results

### Model validation

**Figure 2:**
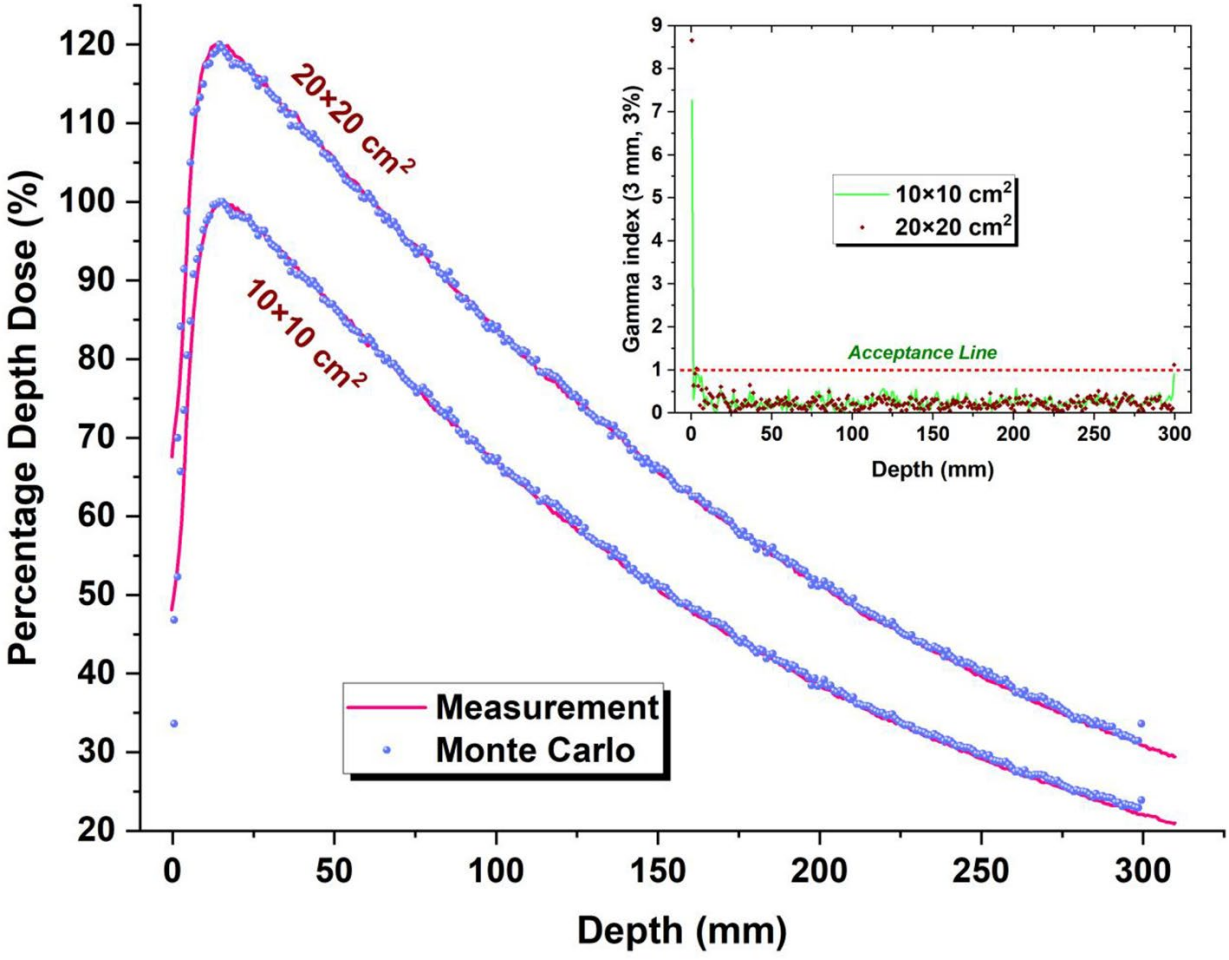
Comparison of calculated and measured percentage depth doses. To separate the data related to 10 × 10 cm^2^ field size from the 20 × 20 cm^2^ field size, the data owned by the larger field was multiplied by 1.2. The inset figure shows gamma passing rate for both fields.

After applying different energy peaks for the electron beam hitting the target, the value of 6.02 MeV was found to be the optimum energy for matching the calculated and measured PDD together in both 10 × 10 and 20 × 20 cm^2^ field sizes. The rate of passing gamma-index for this energy was 99.6% and 99% for 10 × 10 and 20 × 20 cm^2^ field sizes, respectively. Figure 2 shows the comparison of calculated PDDs with measured data using gamma-index function.

**Figure 3:**
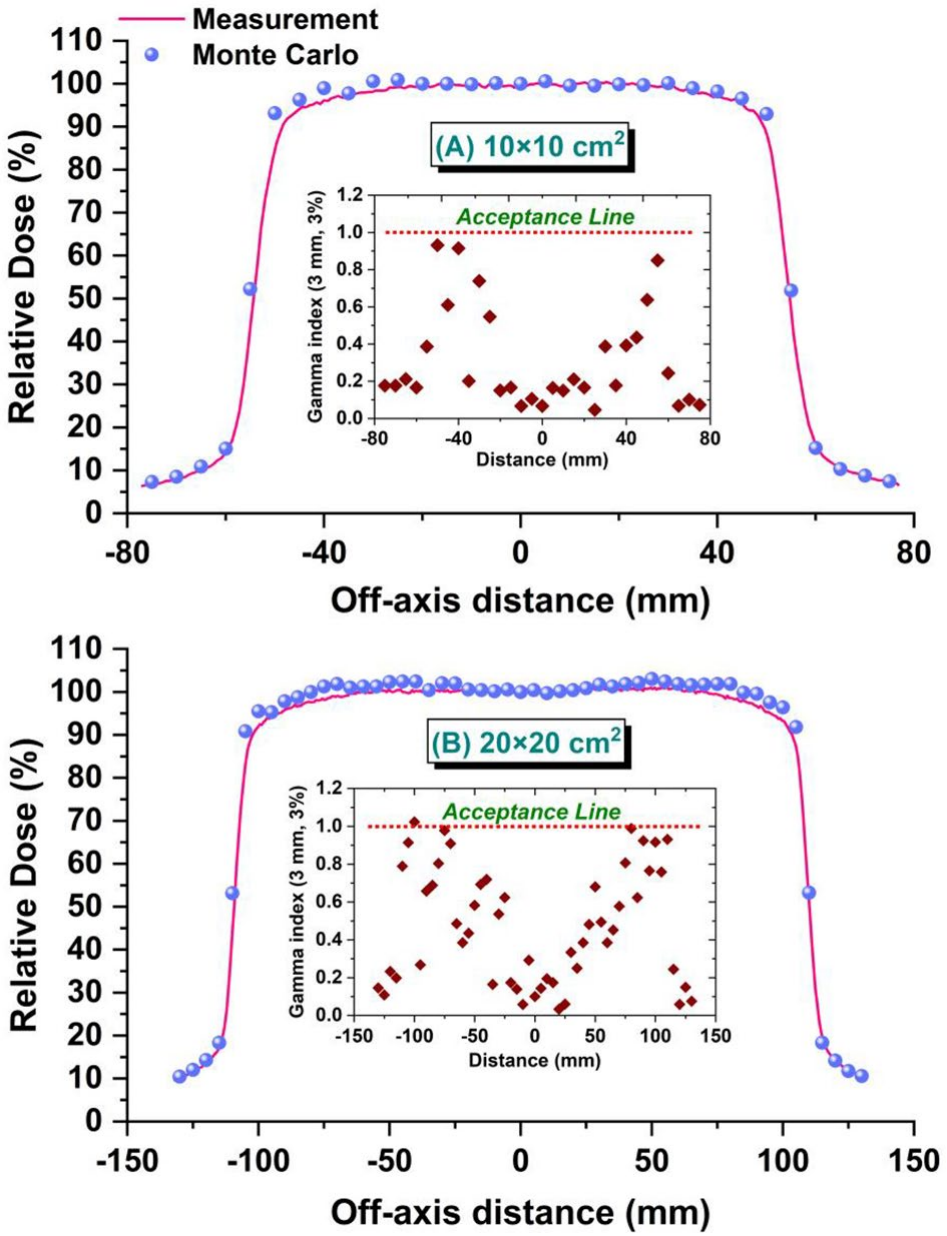
Comparison of calculated and measured dose profiles at 10 cm for treatment fields of (**A**) 10 × 10 cm^2^ and (**B**) 20 × 20 cm^2^. Inset figures represent gamma passing rate of profiles.

To match the calculated dose profile curves with measurement, the FWHM_spatial_ = 1.2 mm and FWHM_energy_ = 1.2 MeV were chosen for the electron beam impinging on the Bremsstrahlung target (Fig. 3). Under this condition, the rate of passing gamma-index for 10 × 10 cm^2^ and 20 × 20 cm^2^ treatment fields was 100% and 98%, respectively.

In the validation process, 2 × 10^10^ electron-histories were traced in the MC simulations to kept all relative errors of calculations related to PDD and profile curves within 1%. However, in a few points, in the tail of profile curves and superficial or deep points in the PDD curves, the relative error of calculations exceeded 1% and reached 3%.

### Dosimetric parameters: grid field vs. open field

**Figure 4:**
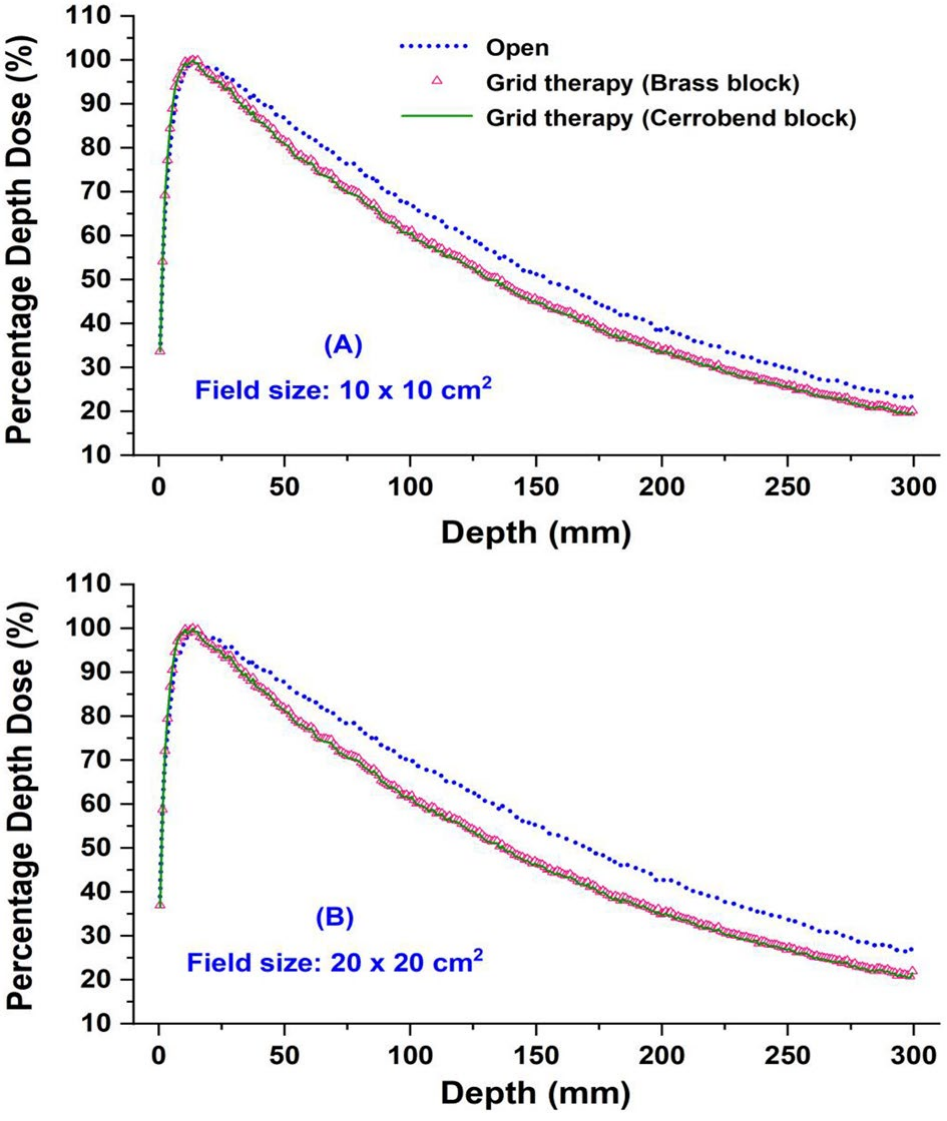
Percentage depth doses for open and grid fields of (**A**) 10 × 10 cm^2^ and (**B**) 20 × 20 cm^2^.

**Figure 5:**
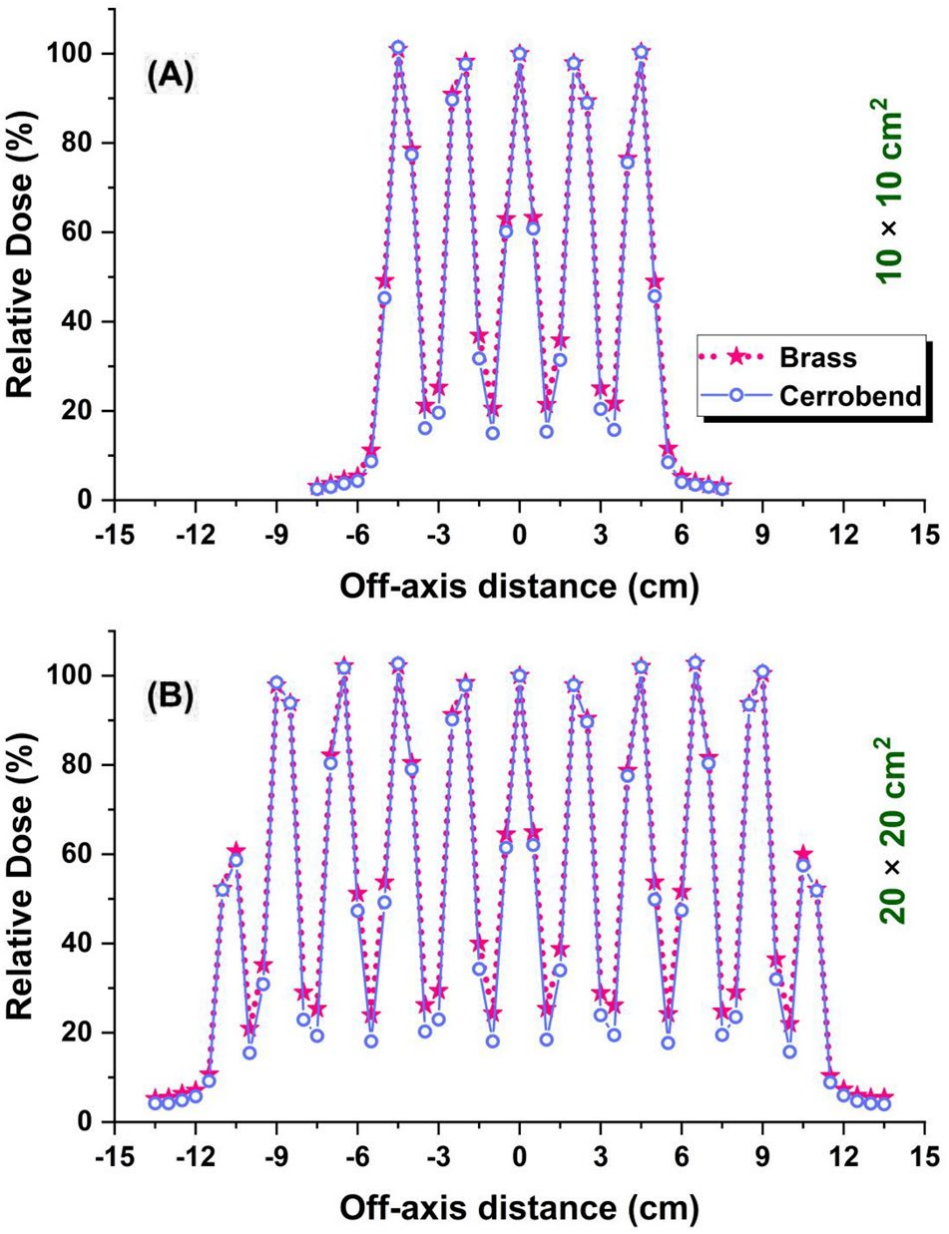
Dose profiles at 10 cm depth inside the water for grid fields blocked by different types of grid (brass and cerrobend) for two field sizes (**A**) 10 × 10 cm^2^ and (**B**) 20 × 20 cm^2^.

The comparison of PDD curves between open and grid fields is shown in Fig. 4. The comparisons were performed for two field sizes of 10 × 10 and 20 × 20 cm^2^. Figure 5 presents the dose profile curves for grid fields of 10 × 10 and 20 × 20 cm^2^. To evaluate the effect of grid genus on the PDD and dose profile curves, the calculations were carried out for two different materials, brass and cerrobend, as the grid material. With tracing 2 × 10^10^ electron-histories in the MC simulations, it was tried to keep the relative error of these calculations within 1%.

Table 2 compares the calculated values for TPR_20/10_, output factor, and d_max_ under the open and grid fields of 10 × 10 and 20 × 20 cm^2^. To evaluate the effect of grid genus on these parameters, the calculations were performed for two different grid blocks (brass and cerrobend).

**Table 2:** Dosimetric characteristics of grid fields compare to open fields. The absolute uncertainties for calculated TPR_20/10_ and d_max_ are 0.009 and 1.0 mm, respectively.

	10 × 10 cm^2^			20 × 20 cm^2^	
	TPR_20/10_	d_max_ (mm)	Output factor	d_max_ (mm)	Output factor
Open field	0.678	15.5	1.000	14.5	1.045
Brass grid	0.648	13.5	0.782	13.5	0.813
Cerrobend grid	0.645	13.5	0.772	13.5	0.790

Table 3 compares the average energy and fluence of photons for treatment field of 10 × 10 cm^2^ and 20 × 20 cm^2^ in open and grid fields. The results were presented at different depths of 1.5, 5.0, and 10.0 cm inside the water phantom. The contribution of primary, scattered, and total photons in the spectra was classified, as well. All statistical checks for MC calculation of photon spectra were passed. Relative uncertainties of MC calculation for photon fluence was kept within 5% in most of the energy bins.

**Table 3:** Contribution of primary and scattered photons in the fluence and average energy of 6 MV photons at different depths inside the water for open and grid fields of 10 × 10 and 20 × 20 cm^2^. The relative uncertainty in fluence calculations was kept within 1%.

Depth	Open field			Grid field						
				Brass grid			Cerrobend grid			
	Primary	Secondary	Total	Primary	Secondary	Total	Primary	Secondary	Total	
Photon fluence (10^8^/cm^2^/MU)										10 × 10 cm^2^ treatment field
1.5 cm	12.89	4.09	16.98	11.46	1.29	12.75	11.43	1.16	12.59	
5 cm	9.74	5.67	15.41	8.67	1.63	10.30	8.65	1.47	10.12	
10 cm	6.62	5.55	12.17	5.91	1.55	7.47	5.90	1.39	7.29	
Photon average energy (MeV)										
1.5 cm	1.83	0.35	1.47	1.90	0.59	1.77	1.90	0.57	1.78	
5 cm	1.92	0.47	1.38	1.99	0.63	1.78	1.99	0.62	1.79	
10 cm	2.04	0.55	1.36	2.12	0.69	1.82	2.12	0.67	1.84	
Photon fluence (10^8^/cm^2^/MU)										20 × 20 cm^2^ treatment field
1.5 cm	13.22	6.94	20.17	11.50	2.31	13.81	11.45	1.93	13.39	
5 cm	10.02	9.80	19.82	8.71	2.92	11.63	8.68	2.50	11.18	
10 cm	6.86	10.36	17.22	5.95	2.96	8.91	5.93	2.56	8.49	
Photon average energy (MeV)										
1.5 cm	1.81	0.28	1.28	1.89	0.57	1.67	1.90	0.52	1.70	
5 cm	1.89	0.35	1.13	1.99	0.54	1.62	1.99	0.51	1.66	
10 cm	2.00	0.40	1.03	2.11	0.54	1.59	2.11	0.52	1.63	

**Figure 6:**
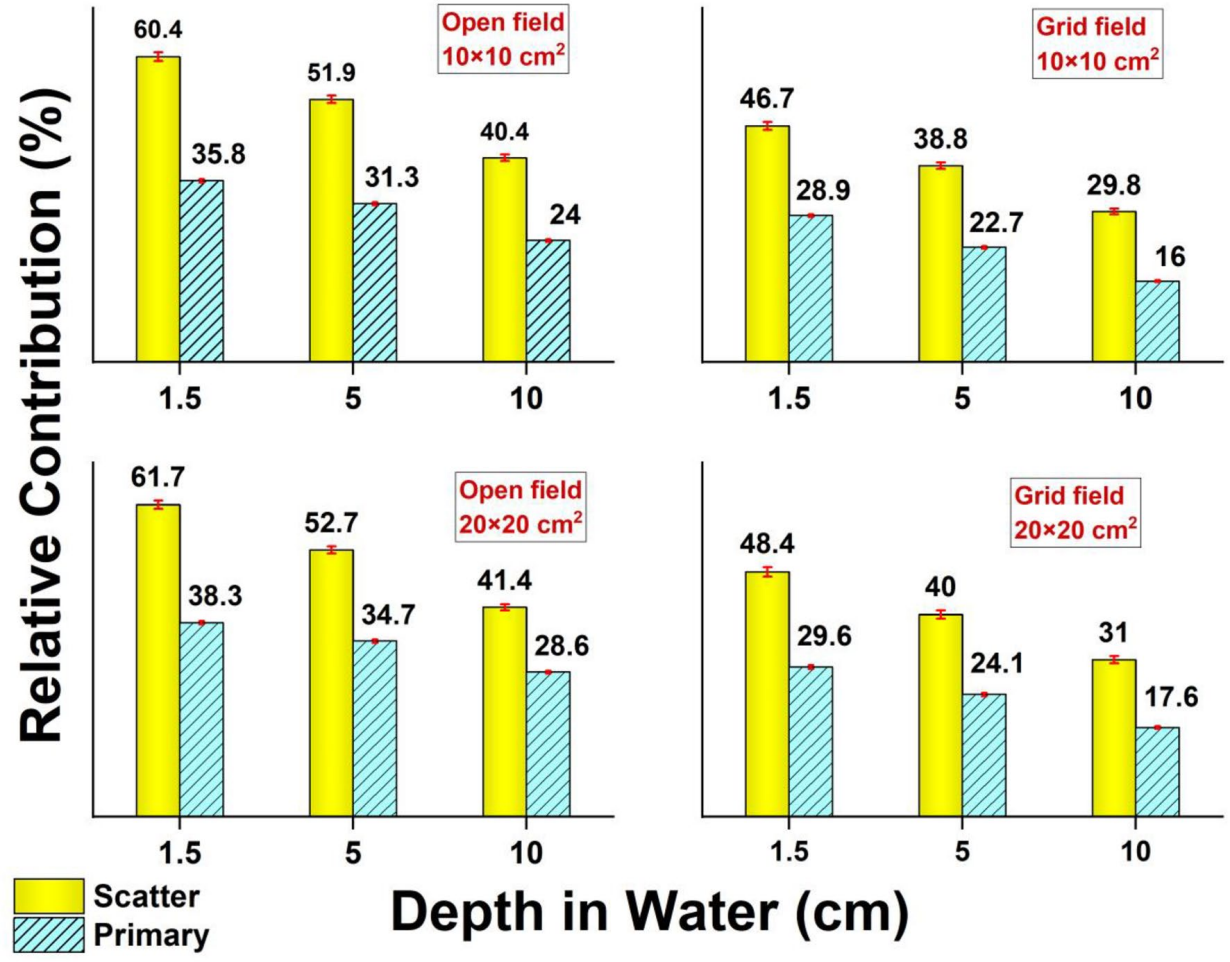
Relative contribution of scattered and primary photons to the depth dose in open and grid. Normalization was performed to the total dose in the open field of 20 × 20 cm^2^.

In Fig. 6, the relative contribution of scattered and primary photons to the depth dose was presented for open and grid fields (collimated by Brass grid). All calculated depth doses were normalized to the total dose in the open field of 20 × 20 cm^2^ (the maximum dose in data).

In the next analysis, in-air calculations showed that total photon fluence inside a 50 × 50 × 0.1 cm^3^ cube is 6.95 and 3.15 (all in 10^7^/cm^2^/MU) for open and grid fields of 10 × 10 cm^2^, respectively. It means, the attenuation power of the grid is nearly 50%.

**Figure 7:**
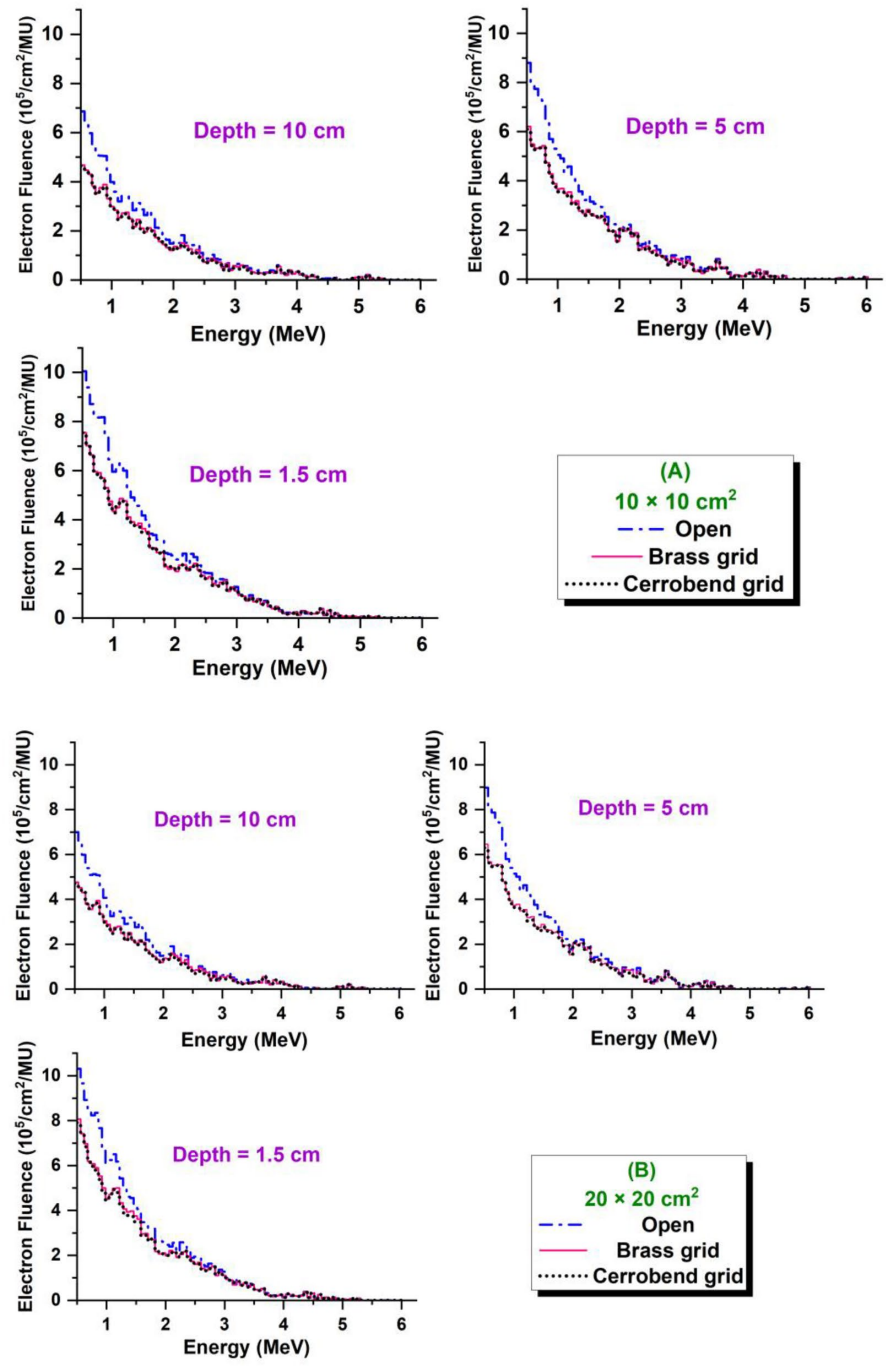
Spectra of electrons produced by 6 MV photons at different depths of 1.5, 5.0, and 10.0 cm inside the water in open and grid fields of (**A**) 10 × 10 cm^2^ and (**B**) 20 × 20 cm^2^.

In Fig. 7, the spectra of the electrons produced by the photons inside the water were shown at different depths of 1.5, 5.0, and 10.0 cm. The results were also presented in details in Table 4 for the open and grid fields of 10 × 10 and 20 × 20 cm^2^. All statistical checks for MC calculation of electron spectra were passed. The maximum relative uncertainty for electron fluence in Table 4 was 5%.

**Table 4:** Fluence and average energy of electrons produced by 6 MV photons at different depths of 1.5, 5.0, and 10.0 cm inside the water in open and grid fields. The relative uncertainties in fluence calculations was kept within 1%.

Field size (cm^2^)	Depth = 1.5 cm			Depth = 5.0 cm			Depth = 10.0 cm		
	Open	Brass	Cerrobend	Open	Brass	Cerrobend	Open	Brass	Cerrobend
Electron fluence (10^7^/cm^2^/MU)									
10 × 10	1.83	1.47	1.46	1.48	1.18	1.15	1.18	0.92	0.90
20 × 20	1.87	1.52	1.49	1.51	1.20	1.17	1.20	0.93	0.91
Electron average energy (MeV)									
10 × 10	1.48	1.56	1.56	1.45	1.52	1.52	1.48	1.54	1.54
20 × 20	1.48	1.55	1.55	1.44	1.52	1.51	1.48	1.55	1.53

## Discussion

Figure 4 shows that independent of field size, the grid genus does not change the PDDs, effectively. However, PDDs in grid fields are significantly (7%) lower than in open fields after the build-up region. The difference in PDDs between open and grid fields is more pronounced in 20 × 20 cm^2^ field size and reached to 10%. Similar findings in the literature confirm the lower PDDs for grid field compared to the open field^5,25^. This finding strengthens the first scenario since it is expected that the grid makes the photon beam softer and consequently the PDDs decrease^26^. To confirm this hypothesis, we were motivated to compare electron and photon spectra at different depths of 1.5, 5, and 10 cm inside the water between grid and open fields.

Dose profile curves calculated for grid fields (Fig. 5) originates from this fact that the dose at the regions positioned directly below the grid holes appear as the peak since they receive a significant portion of primary photons. On the other hand, the regions blocked by the grid only receive the scatter radiation, so these regions appear as the valley. The valley-to-peak ratio is known as the spatial fraction by which the toxicity of high-dose radiation is indicated (the higher spatial fraction, the lower toxicity of radiation). In this study, the spatial fraction for a 10 × 10 cm^2^ field blocked by the brass and cerrobend grid is 0.23 and 0.17 while for the 20 × 20 cm^2^ field, it is 0.26 and 0.20, respectively. The spatial fraction of 0.26 found in this study for the 20 × 20 cm^2^ field is comparable with 0.27 reported by Buckey et al.^25^. As a result, the brass grid with a higher spatial fraction and lower mass density is preferred to the cerrobend grid. From Fig. 5, one can also see that the difference in spatial fraction between the cerrobend and brass grid originates from the deeper valley in the dose profile corresponding to the cerrobend grid because cerrobend has a higher photon attenuation coefficient^37^ compared to brass. The peaks, the dose corresponding to the regions under the holes, do not significantly change between brass and cerrobend grids.

Table 2 compares the calculated values for d_max_, output factor, and TPR_20/10_ under open and grid fields of 10 × 10 and 20 × 20 cm^2^ field sizes, as well. One can also observe that grid genus does not significantly affect other dosimetric parameters. Calculated output factor for open field of 20 × 20 cm^2^ was 1.045 that is well compatible with 1.053% measured by Cho et al.^38^. The results demonstrated that output factors for grid fields are 0.2 lower than in open fields. Additionally, for open fields of 10 × 10 and 20 × 20 cm^2^, the calculated d_max_ is 15.5 and 14.5 mm which matches well with measured data, 15.5 and 13.5 mm, respectively. For grid fields, independent of grid genus, the d_max_ was calculated 13.5 mm that is comparable in a good order with 12 mm and 14 mm reported in the previous studies^5,25,26^. Given the absolute uncertainties of the calculations (1 mm), the d_max_ is the same for open and grid fields. From Table 2, it can be also seen that TPR_20/10_ in the grid field is 0.648 ± 0.009 which is ~ 5% lower than 0.678 ± 0.009 in the open field. The difference in TPR_20/10_ of open and grid fields is observable. Accordingly, TPR_20/10_ is still a good index for the beam quality in grid fields and consequently choose the correct ${k}_{q, {q}_{0}}$ in measurements.

The fluence of primary photons in grid fields only 13% is lower than in open field (Table 3). In contrast, the situation is completely different for scattered photons. The peaks in grid fields is significantly shorter than the open fields. The fluence of scattered photons along the beam axes is 70% lower than in open fields (Table 3). It may be due to the reduction in the photon fluence on the water surface and consequently decreasing the scattering from the water. To confirm this, in-air fluence of total photons before reaching to the water was compared between open and grid fields. The results showed that the fluence in the grid field is nearly 50% lower than in open field. It means the intensity of scattering in the water could be half. Accordingly, a 70% reduction in fluence of scattered photons in water is reasonable. From Table 3, it can be extracted that total fluence of photons at each depth under grid fields is 35%, on average, lower than in open fields.

A new definition can be introduced by the results from Table 3 is percentage depth fluence (PDF) which is similar to definition of PDD:

3 $${PDF }_{(d, r)}=\frac{{Fluence }_{(d, r)}}{{Fluence }_{({d}_{max}, r)}}\times 100$$

where d is the depth and r is the equivalent square field size.

The interesting point is that the PDF_(5, 10)_ and PDF_(10, 10)_ in the field blocked by the brass grid is 11% and 13% lower than in open field, respectively. It is worthwhile to remember that the PDDs for this field is up to 7% lower than in open fields. Similar to PDDs, reduction in PDF of grid fields is more pronounced in 20 × 20 cm^2^ field size. It reaches to 15% and 20% for PDF_(5, 20)_ and PDF_(10, 20)_, respectively. Regarding similar behavior of PDD and PDF, it seems the reduction in PDF of grid fields compared to open fields can be introduced as the reason for the reduction in PDD in grid fields.

On the other hand, Table 3 indicates that for 10 × 10 cm^2^ field size, the average energy of primary, scattered, and total photons at d_max_ is 1.83, 0.35, and 1.47 MeV, respectively. If the field was blocked by a brass grid, these values increase to 1.9, 0.59, and 1.77 MeV, respectively. The value of 1.77 MeV is similar to 1.85 MeV founded by Verhaegen et al.^26^ for an stereotactic radiosurgery field produced by a cone with 1 cm diameter. A similar beam hardening can be also seen for depths of 5 and 10 cm. The maximum beam hardening is related to 10 cm depth with 460 keV. The beam hardening is more pronounced for 20 × 20 cm^2^ field size and increases to 550 keV. Generally, beam hardening can on its own self lead to rising the PDDs of grid fields in comparison with open fields which is in contrast with results of Fig. 4.

Figure 6 indicates that independent of treatment modality, scattered photons have the main contribution to the depth dose. The field size does not change the contribution of scattered photons to the depth dose significantly. Nevertheless, with increasing the depth from d_max_ to 10 cm, one can see a reduction of 20% in scattered dose. Similar behavior can be observed also in the case of primary photons. Figure 6 also shows that the primary and scattered photons in open fields have 12% ± 2% and 8% ± 2%, respectively, more contribution to the dose compared to grid fields. In the case of scattered photons, it is in contrast with public belief who thought that the grid increases the contribution of scattered photons compared to open fields. The authors believe that strong attenuation of photon fluence reaching the water, by grid collimator surface is the reason for the reduction in the scattered components of the dose. In other words, as the fluence of photons reaching the water surface decreases, the intensity of scattering in water will decrease, as well. To confirm this, in-air fluence of photons below the grid was calculated with/without grid collimator. The results showed that the grid collimator has a high attenuation power of 50% and decreases the fluence of photons from 6.95 in term 10^7^/cm^2^/MU for the open field to 3.15 in term 10^7^/cm^2^/MU for circumstances where the grid collimator is mounted on the linac head.

As Fig. 7 shows, independent of treatment modality (grid or open), contribution of 560 keV electrons to the spectra is more pronounced than in other energy bins. With increasing the electron energy, the contribution to the spectrum is continuously reduced so that the fluence of 6 MeV electrons reaches zero. A similar behavior for electron spectra was also founded by Verhaegen et al.^26^ for a circular field with 5 cm diameter. Figure 7 also shows that the grid genus does not significantly change the electron spectrum while, a 20% reduction in depth fluence can be seen in grid fields compare to open fields. However, according to Table 4, the percentage depth fluence of electrons in the open and grid fields are the same. Table 4 also disclosed that the average energy of electrons in grid fields is only up to 80 keV harder than in open fields which is ignorable compared to 511 keV (electron mass). So, lower PDDs in grid fields are not associated with electron spectra in the water.

Due to uncertainties in beam quality and unavailability of dosimetric parameters in grid device, the beam calibration introduces notion of poor accuracy. Additionally, physical devices these days are becoming passe. In such situation, it is prudent to use multi-leaf and create lattice pattern that is easily to transform a complex to simple treatment.

The limitation of this study was determining the fluence component of the photons that scattered from the grid toward the water without any interaction in the water. This limitation originated from the inability of the FT and FU cards in MCNPX. To overcome this limitation, conducting a similar study via the BEAMnrc MC code is recommended. However, simulating the grid block by BEAMnrc modules may be challenging.

## Conclusion

The present Monte Carlo study was devoted to addressing some unknown dosimetric aspects of grid collimator in SFRT. The evidence showed that the brass grid with a higher spatial fraction and lower mass density is preferred to the cerrobend grid. One can also observe that grid does not significantly affect other dosimetric parameters. The results also demonstrated that output factors for grid fields are 0.2 lower than in open fields. The d_max_ is the same for open and grid fields. The difference in TPR_20/10_ of open and grid fields is observable (~ 5%). Accordingly, TPR_20/10_ remains a reliable index for assessing beam quality in grid fields, and subsequently aids in selecting the appropriate ${k}_{q, {q}_{0}}$ values for measurements. This is due to slow function of ${k}_{q, {q}_{0}}$ with beam energy. The main issue was the mystery behind the lower PDDs in grid radiotherapy compared to conventional radiotherapy. To this end, using MC simulation, spectra of electrons and photons at d_max_, 5, and 10 cm depths inside the water phantom were compared between open and grid fields. The evidence demonstrates that the material used for the construction of the grid has no discernible impact on the spectra, and as a result, there is no significant effect on the changes in PDDs. Calculated electron spectra did not show significant changes between grid and open fields to guide us in the explanation of lower PDDs in grid therapy. Nevertheless, photon spectra in grid fields were found up to 550 keV (at 10 cm depth) harder than in open fields which in turn could potentially lead to increasing the PDDs. On the opposite, the percentage depth fluence in grid fields can be even up to 20% lower than in open fields which can result in lower PDDs. The 20% reduction in percentage depth fluence is the reason for lower PDDs in grid therapy compared to the open fields.

### Supplementary Information

Supplementary Information.

## Data Availability

All relevant data are within the paper.
